# Soft infrastructure: the critical community-level resources reportedly needed for program success

**DOI:** 10.1186/s12889-022-12788-8

**Published:** 2022-03-02

**Authors:** Shane A Kavanagh, Penelope Hawe, Alan Shiell, Mark Mallman, Kate Garvey

**Affiliations:** 1 School of Health & Social Development, Faculty of Health, Deakin University, Geelong, Victoria Australia; 2 The Australian Prevention Partnership Centre, 1240 Haymarket, New South Wales Australia; 3 Menzies Centre for Health Policy and Economics, University of Sydney, Sydney, Australia; 4 Department of Public Health, School of Psychology and Public Health, La Trobe University, Bundoora, Victoria Australia; 5 Department of Health, Hobart, Tasmania Australia

**Keywords:** Scale-up, Funding, Resources, Infrastructure, Program costs, Asset development, Implementation

## Abstract

**Background:**

The mechanisms typically used to fund health promotion in communities, either as part of an effort to scale-up programs or to support the design of local activities, often pay insufficient attention to the foundational means of enhancing well-being. Only recently have researchers begun to critically ‘unpack’ how funding processes connect with and activate local community capacities.

**Methods:**

We conducted a thematic analysis of 33 interviews with policy and program administrators in public health and local community workers and volunteers. We invited them to expound on their understandings of resources - specifically, what needs to be in place to make funded programs successful and/or what do communities draw on to make funded programs effective.

**Results:**

Policy and program administrators reflected mostly on the importance of traditional resources, such as adequate funding and staffing. Community-based participants often went further to describe psychological and sociological resources – the “soft infrastructure” which included trust and hope. Both groups emphasised the importance of building networks and relationships at multiple levels. Community workers also provided examples of how resources grow and improve in value in combination with other processes or through pathways of resource use or resource distribution. So, resources like information/knowledge are made more valuable when relayed locally. Physical amenities (e.g., meeting spaces, kitchens) have an instrumental role, but also act powerfully as a symbolic resource for identity. Participants reported that funding processes can damage the resources required for community health improvement. Funding instability undermines capacity. The ongoing threat of funding removal was described by one administrator as community “bullying”.

**Conclusions:**

Processes of health promotion funding, and even standard processes of program scale-up and readiness assessment, risk underestimating the range of resources that are fundamental for community health improvement, particularly among disadvantaged communities. Funders should design ways to resource communities so that there is constant attention to and coaching of critically important diverse processes of resource growth, independent of program-specific funds.

**Supplementary Information:**

The online version contains supplementary material available at 10.1186/s12889-022-12788-8.

## Introduction

On the back of 40 years of investment in the design and testing of interventions we are now in a position where, for 50% of known risk factors, there is an effective policy or program that could be put in place to reduce the disease that results from that risk [1]. Governments now have a strong rationale for ‘evidence-based health promotion’ and in many countries a permanent health promotion workforce exists alongside funding streams for population-based health promotion [2].

However, scaled-up programs frequently fall short of their hypothesised effects [3]. Researchers commonly attribute this to one of two reasons: either to the move to different populations [4] or to locally made, adaptations to the program [5]. We suggest another reason for diluted effects: namely insufficient understanding of the nature of resources required for effective health promotion to take place. The resources considered for scale up in prevention and evidence-based health promotion typically include staffing, training, leadership, technologies/materials [6]. As evidence-based prevention has expanded so has the development of ‘technical assistance systems’ intended to develop human resources, and enhance delivery, quality and sustainability of new programs [7–9]. Indeed by 2015 there were eight implementation frameworks, each drawing attention to how resources might need to be mobilised, organised, and strengthened in order to deliver effective programs efficiently and in ways that match local contexts [10]. However, separate from the external resources that accompany scale-up efforts, program funders possibly underestimate the nature of the local ‘indigenous’ community resources required to “rise up” to meet programs as they are introduced. These are resources not likely to be part of the funding package and their further articulation may require a deeper understanding of the way communities themselves develop local capacities for problem-solving. Resources can be thought of as things that communities need to function effectively and undertake change/improvement [11, 12]. They take a variety of forms, including: people and their knowledge, skills, and relationships; settings, which provide venues for interaction and action; and events, which help to build identity and foster group values [13]. Narratives that a community has about itself may also be thought of as a resource for community health improvement [14].

While there is an established literature in the field of community readiness and its systematic assessment, [15, 16] the focus of readiness assessment is on the interest, engagement, and capacity of the community to implement a particular program. Our interest instead is on core community capacity and factors that may make *any* health or social program effective. Hence the need to understand more broadly a community’s resources and its capacity to solve problems.

We undertook a series of interviews with public health funders/policymakers (government employees and peak non-government organisations) and community workers and volunteers in urban, disadvantaged communities in an Australian city. We sought to observe and understand how resources at a community level are understood and articulated and how they improve or lose value/importance. We did this for two reasons, first to suggest ways to support the development of these resources; and second, to better appreciate the complex interactions between programs and contexts.

## Methods

The study was conducted within the context of a collaborative, chronic-disease-prevention research partnership between government/policy makers and university-based researchers based in Hobart, Australia [17]. Initially, interviewees were purposively selected by an “insider” within the state public health department. The criterion for selection was involvement in the delivery of community-based health promotion policy and programs at either the community or administrative level. Further interviewees were identified through snowball sampling. This process was continued until saturation was achieved [18]. The interviewees were, or had been, involved directly or indirectly with chronic disease prevention policy and programs in Tasmania. They came from multiple organisations including government departments, local government, non-government organisations, community centres and religious groups, and included both employees and volunteers.

Our participant information explained that the purpose of the study was to understand how different types of resources (e.g., funding, skills, knowledge, community groups, community spirit and history) and resource gaps affect how government, health, community workers, and volunteers are able to do their work. In the interviews themselves, to create an easy flow of conversation, we framed this in two ways. We asked interviewees to reflect on why some programs were successful and others were not (e.g., things that needed to be in place, things that might clash or converge). Secondly, we asked about the different things that communities and health workers and volunteers need to draw on for successful community health programs. We tried to fully understand what the participant considered a resource. The interview ended with questions about the effectiveness of current ways in which governments invest in communities in order to appreciate how current means to strengthen community capacity are faring.

Audio recordings were transcribed and analysed with QRS NVivo software. Thematic coding was used to identify emergent themes related to the types and role of resources within interventions [19]. These themes were identified by three authors through on-going discussions during the coding process. An initial coding structure focussed only on resources themselves. However, a subsequent coding structure was developed that identified resources and the processes going on around them.

Interviews were conducted in April/May 2018. The majority of participants (26) were interviewed by two interviewers in tandem with one taking a leading role and the other assisting the flow of conversation. The remaining seven participants were interviewed by a single interviewer. Interviews took approximately one hour and were conducted face-to-face or by phone. Ethics approval for the project was provided by the La Trobe University Human Ethics Office (HEC18018). We used the consolidated criteria for reporting qualitative research (COREQ) guidelines to guide the reporting of our study (see Additional file 1) [20].

## Results

Interviewees easily engaged with the topic of resources and provided a rich dataset from which to address the research questions. There were no refusals. Saturation was reached after 33 interviews. The final sample consisted of 17 policy and program-based administrators and 16 community-based staff and volunteers. Policy and program administrators were involved in development of policy, commissioning and administration of programs and based in a government department, peak bodies, local government, and non-government organisations (hereafter called “administrators”). Community-based participants were current or previous staff or volunteers at local community centres, churches, and community health centres.

There was a large overlap between the perspectives offered by the two groups, but distinctive pictures also emerged. Policy and program administrators reflected mostly on the importance of traditional resources such as people and funding, while those based in the community extended the conversation more into non-traditional resources such as trust, hope and venues/places that create “safe spaces” for social interaction and social development.

### People are foundational resources

The resource that many participants talked about first was people. This was especially so for respondents working in policy and program administration. They spoke of the need for a key person who makes the program work.

“I think (program success) can be quite key person dependent sometimes.” Administrator, ID7.

“The reason why (the program) is great is because it’s got an amazing person that runs that program, probably does a lot more volunteer hours than what they’re actually funded for.” Administrator, ID3.

“Personalities play a big role. […] We call ourselves community leaders. And it’s our role to support and guide our community.” Community worker, ID14.

The right people with time and skills to devote to local community work, paid or otherwise, can be hard to find in disadvantaged areas compared to those with higher socioeconomic status.

(Name of area) is really different to (name of area), where the community just seems more dispersed. It’s more of a rural farming community, less educated, from what I can tell, and there seems to be less people within the community actively setting up those kinds of programs. Administrator, ID9.

Volunteers are vital but administrators are wistful about the frequent circumstance of not having the money to pay volunteers.

“Volunteers […] get to the point where they are burned out. We’ve got a couple of really engaged volunteers who run heaps of sessions and one particular one is a former teacher and she loves sharing information. She does heaps but a few times, she’s been like, “wouldn’t it be nice to be paid?”. Yeah it would.” Administrator, ID7.

Community workers spoke extensively about their role as building people within their communities – their hope, their confidence and their trust. Program activities provide opportunities for personal growth.

“It gives them that, they feel better about themselves, they have more confidence, more able to get up and speak […] and at the end of that thing, they had confidence, self-esteem.” Community worker, ID14.

Community workers also spoke to the fragility of the pathway to hope and how its creation rested on not just providing experiences of empowerment (as above) but the creation of role models and people with good life experience within reach. It was therefore a tenuous task to create hope in a community with harsh life experience.

“Why would you teach your children to have hope if you’ve had experience your whole life of let downs? Because you want to protect your children. So, you don’t set them up to be disappointed, which means you don’t set high goals for education, employment. You keep it to a real practical […] if you make it to the end of primary school and don’t get suspended, it’s a whole different perspective on life. But if you see people around you succeed, or you’ve seen the possibilities, or you can identify opportunities in your life, then you are going to take a different path.” Community worker, ID18.

### Physical space and amenities are essential – on a practical level, but also socially and symbolically

Physical amenities and structures, such as access to kitchens for nutrition/cooking education or meeting spaces, are vital for providing the very basics of conducting programs. Without this, some programs cannot operate at all.

“You actually need a place where people can meet. You (also) need open space, where they cannot feel hemmed in, and they can meet outside.” Administrator, ID11.

But the importance of physical structures goes beyond providing a venue for programs. Structures like neighbourhood community centres create a safe social space, where all people are welcome. Hence, they act as the foundation for building resources on multiple levels.

“(Community centres) give people a place to go that is non-judgmental and services (don’t get) shoved down their throat.” Administrator, ID6.

“This is where everyone’s accepted in this community there is no judgment”. Community worker, ID15.

In disadvantaged communities the quality of the physical structures is often poor and demeaning. So, the renovation of a physical space can act as a symbol for transformation in the community which has meaning at a personal level.

“That process (gives our) community a physical tangible example of something that was rundown and old becoming refreshed and new. Because I’m a big believer that one of the easiest, well the simplest way of bringing about change is physical change in the environment. If you’re wanting to change long term mental health, for example, and you want to change the way people feel about themselves, if you change the environment that they’re actually in, that they’re living in, and they start to see progress, and they start to see things looking better and better and better, it’s really interesting how they identify with that themselves. They start to see that change happening inside of them.” Community worker, ID19.

And, on the simplest of levels, the confidence to try something new - like the cooking skills needed to use nutritious ingredients derives from the creation of safe space psychologically and socially. A former community worker (recently turned program manager) explained how she and her staff originally made a lot of wrong assumptions about working with communities. They would just walk in and say “*Here’s your recipe card. Here’s your group. Go!*“ She and her team learned just how distant residents were from being able to take part. She found that trying something new takes personal courage and only happens with trust, support and encouragement.

“We had a lady break down into tears because I handed her a Kiwi fruit and asked her to chop it up and put it in the fruit salad, and she had no idea what it was, she didn’t know what to do with it. She had a complete meltdown, walked away from the program for a week, and then actually came back, and all the people that have supported her previously just said that was amazing that she came back, and she actually stuck at it for six weeks […] her progress has been really slow. But she’s maintained that engagement with the program, and she does want to learn.” Administrator, ID20.

### Relationships are also a vital part of a community’s capacity to engage with programs

At the community level, building relationships is foundational to practice.

“It’s getting to know the people. It’s about building those relationships.” Community worker, ID28.

Relationships between central and local community organisations can determine the pace of action.

“I think the relationships are key, (because) the pace at which you try to do something, I think, is really important. It’s almost like, if you don’t have the foundational relationships, so that the people who are volunteering their time and their resources feel valued, (then) it’s not worth going anywhere, I’d say. So sometimes you have to go slow ’cos you need to build those (relationships).” Administrator, ID3.

Especially when funds are scarce, inter-organisational relationships permit coordination of activity amongst different groups working on the same issues. And resources can be usefully put towards fostering coordination.

“We’re talking about very small investments here (so) it could have been a disaster because if they all went off and did their own thing.” Administrator, ID5.

### The value of resources is multiplied, locally, in a number of ways

The chance that a resource such as information will be listened to and made use of is increased by passing through networks. Local voices are more valuable than outside experts and practitioners find ways to coach messages through local networks.

“You know in the old days we used to send a dietician in to give a talk at a young parents’ group in a community house. We would very quickly realise that probably wasn’t making that much difference in terms of whether somebody was actually receiving and wanting to do anything with the information we provided. Whereas if it’s coming from their networks and peers at the right time and at the right place then it’s much more likely to result in sustainable action.” Administrator, ID5.

“The “who” (in conveying information) is really interesting, […] if it comes from a fellow parent or community member, then it has a lot more value than me as a professional would ever give.” Community worker, ID27.

The value of other resources is also improved through their use. Physical resources risk becoming ‘white elephants’ if not used, so, events and activities harness physical resources in order to create connection and value.

“Of course, there are all the traditional resources in a community that are sometimes under-utilized too. But you know, like physical infrastructure that a community has that often is not used. But often activation of that is through these relationships.” Administrator, ID4.

Health workers can enhance the value of health promotion activities according to local context. For example, in community contexts that are disadvantaged, services and activities are free (in order to maximise the chance of community use). By contrast in more well-off communities, asking for a small fee increases the perceived value of the activity:

“I’m always trying to make things cheaper, and (co-worker) is always trying to make them more expensive! And he says, “You got to give your program a worth.“ If you’re giving it for free, they don’t think it’s worth anything. So, building a value around the program so people know that it’s a good program. And then they will pay for it. I really think that having them chip into the program, which is what they did at (place) worked. They did ask for gold coin donation. If you could afford it. (It) does build a value into that program. So that they feel like there’s a worth. Putting some coin in. And you know, I’ll get something out of it.” Community program volunteer, ID25.

Another way the value of resources – like inter-organisational relationships - is enhanced is by the status or kudos of the participants involved. People with senior status in the community (by virtue of their ‘rank’ in their organisation) pay others a compliment by being out and about in the community and mixing. This also has the advantage of appearing as if the organisation is “big” enough to do so, when in fact it might still be vulnerable and growing its own membership.

“And during the first year, […] one of the community liaison workers said to me, ‘I’m really, really impressed at how involved (name organisation) is in the community.’ I laughed on the inside, because I was thinking: Well, the only person in our (organisation) at this point in time that is involved in the community is me. And all I do is go to meetings and open the venue up. But what (that) really drove home was the power of just being at stuff, and the way people perceive that. People were thinking because the (me, the senior person) at (named organisation) is involved in all these committees […] ‘he’s doing stuff for free, wow, the (organisation) as a whole is really involved…’ And all you’ve done is just turned up at the same place that they are.” Community worker, ID19.

These descriptions provided insight into constant processes of adjustment, building, adding and combining resources that occur as part of community development and strengthening.

### Funding processes can cause harm

We found community health enablement to be a painstaking, dynamic, and delicate process. There was an outpouring of concern about funding processes often acting to undermine rather than strengthen communities. In the first instance, funding has to be sufficient to prevent loss of people/staff through turnover. Otherwise, it breaks the ability to create long term relationships and trust.

“We’ve been running that program for nearly 20 years, but again, it’s been pathetic amounts of money that we’ve given to it.” Administrator, ID1.

“People are the core. […] When your funding doesn’t support that commitment of people, a lot of the projects have people turning over, they’re changing positions, […] their funding’s gone from different parts. They’ve had to drop out […] (so) I think that’s one of the key things: the generosity of resource to let people stay in something long term.” Administrator, ID13.

“Building trust, building relationships, coming together to agree on what are we working for, takes time. Mostly our projects […] funded by government even […] don’t allow for that complexity and relationship building.” Administrator, ID6.

Funding instability undermines capacity. The ongoing threat of its removal is like bullying.

“And (we’ve) also threatened them almost every year that we may not have the money next year. […] every year we’ve said ‘Oh we don’t know if we’re gonna have the money next year.’ I mean it’s absolutely awful. As far as I’m concerned we’re bullying the community sector. That’s a tough word, but I actually believe that we have bullied the community sector by not being clear that this is a program we really value and nurture, they’re doing everything they can within their power to do a good job of it, and yet we threaten them every year that we may not have any funding.” Administrator, ID1.

Time-limited funding undermines the foundations for health improvement. It erodes trust. The logic that health promotion activities can and should become self-sustaining was also questioned. There was also evidence that local government refuse to engage with funding rounds as the costs outweigh the benefits.

“We tend here at Council to shy away from government funding just because we can’t rely on it… It’s like we start this project to set something up, and now what? We’re gonna be left with a project that was great for 6-7 months …” Administrator, ID13.

“I’m pretty cynical about the idea that most programs can be self-sustaining without some level of investment […] I think governments […] are trying to find ways to cut costs, and part of the way to cut costs is by not providing ongoing funding to something, even when there’s an ongoing need.” Administrator, ID3.

Accountability processes and evaluation (as currently designed and performed) are topic focussed and work counter to processes of more generic community strengthening. They distract from telling the story of what really matters.

**“**In order to really liberate more community capacity, there does need to be time and emphasis given to building trust and relationships. […] So, we’re actually doing harm now, currently, with counting widgets and outputs.” Administrator, ID16.

“So yeah, […] what I think is needed is less rigor or rules around funding programs. Maybe like, keeping focused on the big picture of this funding is about wanting to reduce chronic conditions, but having flexibility to experiment and see what works and what doesn’t. Or even (provide funding) to understand what the problem is.” Administrator, ID 17.

The interview prompted one community worker to conclude that funding systems for community health promotion need to be rethought.

“If the government could identify what actually makes for a healthy community, I just don’t think it would necessarily be some of the stuff that we fund at the moment. I think (they would fund instead) some of the stuff that we’ve been discussing this afternoon about creating reasons for the community to get together, helping build relationships, and networks from house-to-house.” Community worker, ID19.

## Discussion

This study set out to uncover what makes funded programs successful at a community level and to identify some dynamics about how resources gain or lose value. We drew on the expertise of those working directly and indirectly with community programs. We identified a broad range of resources, some more visible than others. We acknowledge that our study has limitations. Our study took place in one Australian state. So we are unsure how typical is the experience of frustration expressed with funding constraints and structures, because health promotion organisational structures and funding are inconsistent across jurisdictions.

Overwhelmingly, trust lay at the heart of successful community betterment. Trust and success are eroded by funding insufficiency, but more particularly by funding instability. The constant threat of funding removal was even described by one person as bullying. Trust and hope were talked about as key assets - key outcomes of processes of “building people” and vital for creating confidence for those first forays people may make to learn new life skills - like healthy cooking. This was evidenced in the kiwi fruit story where, a woman followed her first instinct and walked out of a class, rather than stay and feel embarrassed or humiliated by not knowing what a kiwifruit was and how to cut it up. But she returned and completed the six-week course, because she felt supported to do so. Indeed, without confidence and trust, we were told that people will not even dare to develop hope for a better life for themselves and their children. To avoid disappointment, people will make life choices devoid of too much aspiration.

Relationships (workers with community, agency-to-agency, and among local residents) create pathways for action. Inter-organisational relationships permit coordination of activity, which is especially important when formal funds are scarce. Relationships prompt and nurture resources, like the number of volunteers and the time they give. Some communities have more volunteers than others. Communities with more disadvantage can struggle to produce volunteers who can connect and provide support to health promotion programs. The right people and “personalities” are critical, however, ultimately for anyone, burnout is a danger.

The findings support the extensive literature which emphasises the role of cognitive, emotional, social, and relational resources in supporting processes social change [14, 21–23]. Others have called this “soft infrastructure” [24]. We reviewed literature on this previously [25]. In brief, soft infrastructure includes much of what we observed here such as relationships, safe spaces [26] trust and hope, [27] self-efficacy, [28] and world view [14]. A dictionary defines infrastructure as basic physical and organisational structures and facilities (e.g. buildings, roads, electricity) needed for the operation of something [29]. Correspondingly, we observed the need for basic physical amenities for a program to be delivered along with core staff, paid or otherwise. But by prompting participants to consider what makes funded programs effective (or not) we also learned about trust, hope, self-efficacy and relationships. Anthropologists consider the definition of infrastructure to include the social norms and exchanges that are embedded in material forms [30, 31]. The contrast between these two definitions possibly explains why it is that recommended resources for program scale-up and implementation typically includes those aspects that are most visible (staff, venue costs, training) [6]. But those guidelines fail to identify underlying social resources and capacities that also need to be in place to make the infrastructure truly ‘work’ [31].

Many of our results, would be no surprise to scholars or practitioners in the field of community development. Community-based network and empowerment approaches to health promotion have a long history [32, 33]. More recently, asset-based approaches to health promotion have come into prominence [34]. So why is it important to ‘rediscover’ the importance of community-based resources now? This recognition is imperative now because conventional ways to fund and develop health promotion at a population-level risk paying insufficient attention to the primary foundational means of enhancing well-being and behaviour change. Our results suggest that for communities experiencing chronic disadvantage it is not possible to, say, take an interest in what people eat or how much they exercise until one understands *how people live.* Understanding how people live prompts a commitment to creating conditions that help people develop confidence to envisage a different future for themselves, a future which is, of course, much more than what they eat.

Yet, increasingly, the era of evidence-based health promotion requires a focus on particular programs delivered to particular standards [35]. Staffing and materials are focussed on program implementation. It is common in scale-up for there to be insufficient resources for program implementation, [36] let alone, resources for the type of staff and time to develop the soft infrastructure that might be needed from the start. In the US, the field of technical capacity building for evidence-based prevention sprung up to accompany federal funding for the states to deliver evidence-based prevention [7, 8]. It has been operating for more than a decade. Researchers have documented the conflict between faithful adherence to the program dissemination agenda versus community capacity building e.g., working with local organisations in a more basic way, such as to help them develop the accounting systems to make them eligible for funding [37]. However, neither scale-up planning, nor technical capacity-building, go so far as to touch the type of resource development that we describe here i.e., the additional tailored need for personal development (e.g., self-confidence, self-esteem, social relationships, etc.). Yet person-and-network focussed development would be the central agenda if health promotion funding was dominated by a primary interest in community capacity, and secondary interest in the delivery of particular programs, instead of the other way around. Indeed, even in the presence of vast amounts of projects funds, researchers have recently shown that the people-processes, rather than the funds themselves, are the key to community betterment [38].

Other researchers have expressed a similar frustration with the gap between the in-built expectations and assumptions of health systems and what is really needed to engage communities with health interventions. Writing from a rural and remote context in Australia, Jones and colleagues argue that the push for “health literacy” skills in individuals should be balanced with a push for “community literacy” skills among health personnel [39]. There needs to be stronger attention to the intangibles - “trusting relationships, relationship continuity, consistency and credibility” necessary to create community engagement in health ([39] p.4). They outline a set of principles for the development of “civically informed and socially accountable” health systems to enhance capacity to respond to community needs ([39] p.4). None of our interviewees would likely disagree with these principles, but they feel constrained by funding systems that do not fully allow them to act how they would wish.

One key insight from our findings is that resources used for health improvement and program success act in critical combinations. They also grow or diminish through human action/inaction. At the basic level resources need to be supplied - like meeting rooms or kitchens. But they also need to be nurtured and managed. We were told the story of how renovation of shabby buildings provides a powerful symbol for people to identify with - “something run down becomes refreshed and new.” That process of transformation or improvement in value happens also with other resources, like information. Professional educators, dropped into communities to give talks, have little impact. But if the same information comes from a fellow parent or community member then it gets listened to. Hence the value of the resource seems to increase as it passes through local social networks, an idea previously put forward by Hawe and colleagues [40]. This warrants further investigation.

In addition to information being made more worthy through local voice, we had the story of events being enhanced in perceived value by virtue of who attends them. Local people with high status attending events give the events gravitas. This is another example of soft infrastructure adding value. Interestingly, we were told that in some communities, paying for something (a one or two dollar optional fee) increases attendance at activities that would otherwise be free. In other communities, activities must be free for people to attend. Insights and details like these can only come from paying attention and being there long enough to know a community well.

## Conclusions

The chief implication of our study is that priming funds (to prepare communities to be ready for particular programs and able to help deliver them) and implementation funds (to deliver programs themselves) cannot substitute for the primary commitment of funds for staff to be in communities, knowing them, and helping to grow their diverse human and social resources - the soft infrastructure *independent of any particular program*. Ground-level processes take time and flexibility. Local networks require building or curating. We cannot and should not allow populations to lose opportunities to benefit from funded, evidence-based programs. However, if the local ‘receptor capacity’ is not there, if people are not quite at the stage of envisaging a better future for themselves, then a more foundational investment in health development is vital.

## Supplementary Information

**Additional file 1.**

## Data Availability

The dataset is not available for further analysis due to information that could compromise research participant consent and privacy. Consent was specific to the use of data within the research partnership described to participants and specific to the study purpose.

## Abbreviation

ID: Identity
