# Supplementary material for: Soft infrastructure: the critical community-level resources reportedly needed for program success
Source: BMC Public Health. 2022 Mar 2;22:420. doi: 10.1186/s12889-022-12788-8 (PMC8889705; doi:10.1186/s12889-022-12788-8)
Supplement: Supplementary file 1 — Additional file 1. [file 12889_2022_12788_MOESM1_ESM.docx]

**Soft Infrastructure: the Critical Community-Level Resources Reportedly Needed for Program Success**

COREQ checklist

Shane A Kavanagh, Penelope Hawe, Alan Shiell, Mark Mallman, and Kate Garvey

| **Additional File 1.** Additional information as per the Consolidated Criteria for Reporting Qualitative Research (COREQ) checklist | |
| --- | --- |
| Domain 1: Research Team and reflexivity | |
| Personal Characteristics  Interviewer/facilitator; 2. Credentials; 3. Occupation; 4. Gender; Experience and Training | Semi-structured interviews were conducted by SK and MM, who both identify as male. SK has 9 years’ experience in public health research. This was his first qualitative research project. MM is a sociologist and has more than 6 years’ experience in qualitative research. PH identifies as female and is a public health researcher with 30+ years’ experience. AS identifies as male and is a health economist with 30+ years’ experience. KG identifies as female and has extensive experience working in public health and is currently a manager for a state government public health service. |
| Relationship with participants  6. Relationships established; 7. Participant knowledge of the interviewer; 8. Interviewer characteristics | The research project was undertaken via a partnership between the Department of Health, Tasmania and The Australian Prevention Partnership Centre. The Department of Health, Tasmania is a funding partner of the Australian Prevention Partnership Centre. KG is an employee of the Department of Health with a direct role in supporting and monitoring the funding of community health projects, some of which were included in the study. SK, MM, PH and AS were university-based researchers. SK and MM were employed with funding from the Australian Prevention Partnership Centre.  Only the two interviewers (SK and MM) had full knowledge of the identities of the participants. All participants, except one, were previously unknown to the interviewers. Participants were assured that their anonymity would be maintained throughout the research process by the research team. Only SK, MM, PH, and AS had access to the de-identified transcripts. KG did not have access to any of the primary data, so as to protect the anonymity of the participants.  Study participants were informed that the interviewers were working in partnership with the Department of Health, Tasmania. All authors were named on the study consent form. |
| Doman 2: Study design | |
| Theoretical framework  9. Methodological orientation and theory; | This study was co-designed with KG at the Department of Health, Tasmania. We designed and conducted interviews and adopted a grounded theory approach. |
| Participant selection  10. Sampling; 11. Method of approach; 12; Sample Size; 13. Non-Participation | Participants were purposively sampled. The criterion for selection was involvement in the funding or delivery of community-based health promotion programs at either the community or administrative level, using information available through Dept Health funding records. Further interviewees were identified through snowball sampling after the completion of participant interviews. |
| Setting  14. Setting of data collection; 15. Presence of non-participants; 16. Description of sample | Interviews were conducted in private both at Department of Health offices and in community-based locations, including local government offices and community centres. No non-participants were present during interviews.  The final sample was comprised of policy and program administrators and community-based staff and volunteers working across 10 communities. Policy and program administrators were involved in the development of policy, commissioning, and administration of programs and based in a government department, peak bodies, local government, and non-government organisations. Community-based participants were current or previous staff or volunteers at community centres, churches, and community health centres. |
| Data collection  17. Interview guide; 18. Repeat interviews; 19. Audio/visual recording; 20. Field notes; 21. Duration; 22. Data saturation; 23. Transcripts returned | Interviews were guided by a pre-prepared interview protocol. The interview was semi-structured to allow participants to openly convey their perspectives and to elaborate on particular areas of interest. Interviews were around 60 minutes in length and were audio recorded for later transcription. Participants were recruited until data saturation had been reached. The interviewers undertook extensive discussion of the interviews after each of the joint interviews and later discussed their observations via email and in person. |
| Domain 3. Analysis and findings | |
| Data analysis  24. Number of data coders; 25. Description of the coding tree; Derivation of themes; 27. Software; 28. Participant checking | PH developed an initial pilot coding structure based on an analysis of early transcripts [Corbin J, Straus, A. *Basics of qualitative research: Techniques and procedures for developing grounded theory*: Sage publications. 2014] in consultation with SK and MM.  The coding structure was refined by PH, SK and MM and transcripts were coded using NVivo qualitative data management software. A report of study findings was disseminated to participants prior to the write up of this paper. It was favourably received in terms of accuracy of themes and issues captured. |
| Reporting  29. Quotations presented; 30. Data and findings consistent; 31. Clarity of major themes; 32. Clarity of minor themes | Findings are supported with quotes extracted from interview transcripts from multiple study participants. Consistency between data and findings was iteratively checked throughout the analysis to ensure consistency and accuracy between the two. |
